# Human and Chimpanzee Gene Expression Differences Replicated in Mice Fed Different Diets

**DOI:** 10.1371/journal.pone.0001504

**Published:** 2008-01-30

**Authors:** Mehmet Somel, Hilliary Creely, Henriette Franz, Uwe Mueller, Michael Lachmann, Philipp Khaitovich, Svante Pääbo

**Affiliations:** 1 Partner Institute for Computational Biology, Shanghai Institutes for Biological Sciences, Chinese Academy of Sciences, Shanghai, China; 2 Max-Planck-Institute for Evolutionary Anthropology, Leipzig, Germany; 3 Center for Biotechnology and Biomedicine, University Leipzig, Leipzig, Germany; Temasek Life Sciences Laboratory, Singapore

## Abstract

Although the human diet is markedly different from the diets of closely related primate species, the influence of diet on phenotypic and genetic differences between humans and other primates is unknown. In this study, we analyzed gene expression in laboratory mice fed diets typical of humans and of chimpanzees. The effects of human diets were found to be significantly different from that of a chimpanzee diet in the mouse liver, but not in the brain. Importantly, 10% of the genes that differ in their expression between humans and chimpanzee livers differed also between the livers of mice fed the human and chimpanzee diets. Furthermore, both the promoter sequences and the amino acid sequences of these diet-related genes carry more differences between humans and chimpanzees than random genes. Our results suggest that the mouse can be used to study at least some aspects of human-specific traits.

## Introduction

Genome sequences from humans and closely related primate species are collected at an increasing rate with the hope of gaining insights into the genetic underpinnings of the human phenotype. However, beyond DNA sequence differences between humans and other primates, such as the chimpanzee, these species experience large environmental and cultural differences. This raises the question of how much of the phenotypic differences observed between humans and other primates are caused by such non-genetic differences.

One example of a cultural difference between humans and chimpanzees is diet. The human diet, despite a multiplicity of local idiosyncrasies, consistently differs from those of other primates in such aspects as high caloric and protein content as well as cooking, *i.e*. heat processing prior to ingestion [1]–[6]. It is plausible that different diets result are correlated with physiological states in humans and chimpanzees and that such states may be physiological responses in the individual to different dietary contents as well as genetically fixed evolutionary adaptations to dietary differences (e.g. see [4], [7], [8]). That diets can cause genetic adaptations is illustrated by lactase persistence in dairying populations [9] and higher copy numbers of the amylase gene in groups consuming starch-rich foods [10].

Although gene expression differences between humans and chimpanzees in multiple tissues have been described [11]–[14], the role of dietary differences on these expression differences awaits investigation. Generally, the gap between genomic and phenotypic features is particularly difficult to bridge when studying traits fixed among humans, since most experimental approaches cannot be applied to humans or higher primates. This leaves model organisms, such as rodents, as one of the few tools available where functional manipulations may allow differences between humans and other primates to be analyzed with respect to their environmental or genetic causes.

Here, we use laboratory mice to analyze, first, to what extent human and chimpanzee diets induce differences in gene expression, and, second, whether such differences may be similar to gene expression differences seen between humans and chimpanzees. Finally, we show that the rate of evolution of genes affected by diet in both the rodents and the primates is higher than for average genes in the human and chimpanzee genomes.

## Results

We fed four groups of six 8-week-old female mice one of four diets *ad libidum*: first, the mouse pellet diet on which they were raised; second, a diet consisting of vegetables, fruit and yogurt identical to the diet fed to chimpanzees in our ape facility; third, a diet consisting of cooked food eaten in our Institute's cafeteria; fourth, a diet consisting exclusively of McDonald's fast food (Table S1).

**Figure 1:**
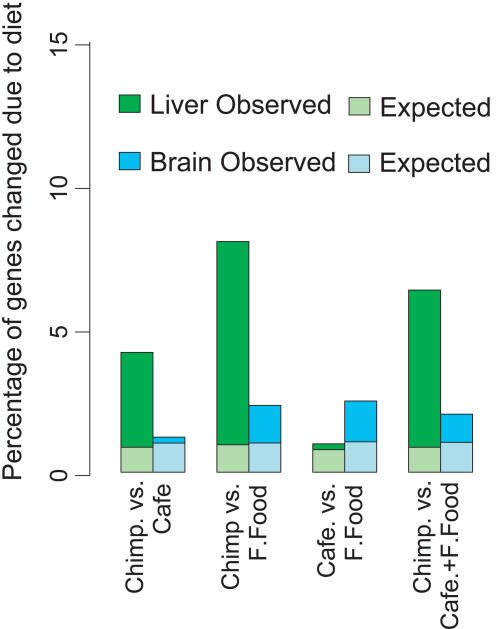
The effects diet on gene expression in mice. The height of each column indicates the percentage of genes showing expression differences (at ANOVA p<0.01) between mice fed two different diets, in liver (green) or brain (blue). The lighter coloured stem of each column shows the percentage of diet-related genes that would be expected by chance alone, calculated by means of 1,000 permutations. The labels are: Chimp-chimpanzee diet; Cafe-human cafeteria diet; F.Food-human fast food diet; Cafe+F.Food-human cafeteria and fast food diets together.

After two weeks, we examined gene expression in liver and brain. Using an ANOVA and permutation test, we find significant expression level differences among mice fed the four diets in liver, but not in brain (one-sided permutation test *p*<0.001 and *p* = 0.16, respectively; Table S2). Similarly, when the effects of particular diets on liver gene expression are compared, all pairs of diets show significant differences from each other (one-sided permutation test *p*<0.02) with one exception: The cafeteria and fast food diets are indistinguishable in terms of liver gene expression (*p* = 0.14; Table S2). We therefore decided to treat these two human diets together. We find that when the human diets are compared to the chimpanzee diet, 830 of the 13,168 expressed genes, or 6.3%, are affected in the mouse liver (one-sided permutation test *p* = 0.030; Figure 1; Table S3).

**Figure 2:**
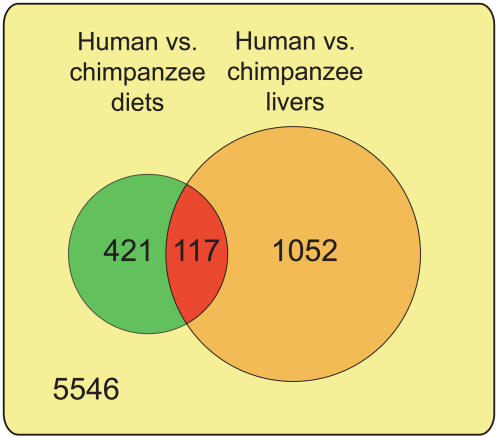
Overlap between liver gene expression differences in mice and primates. The numbers of human-mouse orthologous genes differentially expressed (at ANOVA p<0.01) between mice fed human diets and a chimpanzee diet in liver (green circle), and genes differentially expressed (at t-test p<0.01) between human and chimpanzee livers (orange circle). The number in the overlap between the two circles (red) indicates genes showing significant expression differences in both data sets. A total of 5,546 genes with detectable expression in both data sets show no significant expression differences in either data set.

We then compared these genes affected by human and chimpanzee diet differences in mouse to the 1,169 orthologous genes that differ in expression when human and chimpanzee livers are compared [13]. 10%, or 117 of the genes differentially expressed between human and chimpanzee livers were also among the genes affected by human and chimpanzee diet differences, a proportion larger than expected by chance (one-sided permutation test, *p = *0.001; Figure 2). Thus, using mice fed just two distinct human diets and one chimpanzee diet, it is possible to replicate some of the expression differences observed between humans and chimpanzees. By contrast, expression differences between the original mouse pellet diet and the two human diets (8.9% of genes; Table S3) did not overlap significantly with expression differences observed between human and chimpanzee livers (Table S4). Since mouse pellets, unlike the model chimpanzee diet, have high caloric and protein content and are heat processed, the gene expression differences between humans and chimpanzees seen also in mice fed chimpanzee and human diets (Figure 2) are likely to reflect effects induced in the liver by components of the human and chimpanzee diets, respectively.

A total of 117 genes are differentially expressed both between mice fed human and chimpanzee diets and between humans and chimpanzees in liver (Table S5). We find that these 117 putatively diet-related genes have higher absolute effect sizes (mean differences between groups in units of standard deviation) for human-chimpanzee expression differences than 1,052 non-diet-related genes differentially expressed between human and chimpanzee livers (one-sided Mann-Whitney U test *p* = 0.022). In other words, diet-related genes exhibit larger expression divergence than most differentially expressed genes in liver. 92 of these 117 genes (78%) show up-regulation under the human diet compared to the chimpanzee diet in mouse, but interestingly, there is no significant correlation between the direction of change in mouse under the two diet conditions and the change seen between humans and chimpanzees (Fisher's exact test *p* = 0.6; Table S6; Figure S1). In terms of their functional roles, the 117 diet-related genes are significantly overrepresented in seven biological process categories in the Gene Ontology [15] compared to other genes differentially expressed between human and chimpanzee livers. Notably, five of these categories are involved in metabolism in a broad sense (Table 1). Furthermore, using orangutan gene expression data as an outgroup [16], we observe that the expression levels of these 117 genes are more similar between orangutan and chimpanzees than between orangutan and humans than is the case for other genes differently expressed between human and chimpanzee livers (one-sided permutation test *p = *0.047; Table S7). This would be expected if the effects of the chimpanzee and the orangutan diets were more similar to each other than either were to the effects of the human diets.

**Table 1:** Biological processes significantly enriched in genes potentially involved in human-chimpanzee dietary differences.

Gene Ontology category	# Diet-related genes (total 87)^a^	# Non-diet-related genes (total 711)^b^	*p*-value for enrichment^c^
Vitamin metabolism	3	1	0.005
Sodium ion transport	3	1	0.005
Amino acid biosynthesis	4	2	0.002
Positive regulation of transcription	4	4	0.007
Amino acid and derivative metabolism	11	17	0.006
Carboxylic acid metabolism	15	32	0.003
Organismal physiological process	17	63	0.003

a The number of human genes with mouse orthologs showing expression differences between humans and chimpanzees and between mice fed human and chimpanzee diets in liver, and are found within the relevant GO category.

b The number of human genes with mouse orthologs showing expression differences between humans and chimpanzees in liver, but not between mice fed human and chimpanzee diets, and are found within the relevant GO category.

c Hypergeometric test *p*-value for the GO category being enriched in diet-related genes relative to control genes.

**Figure 3:**
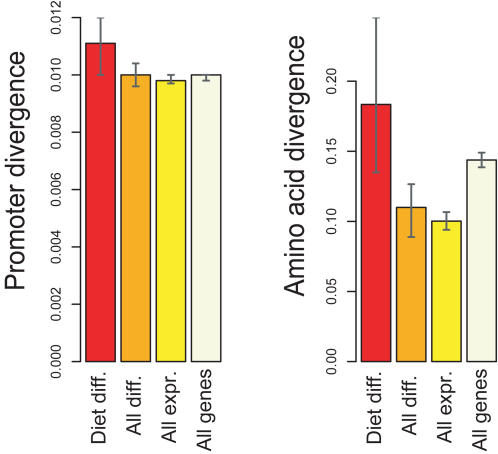
Sequence divergence of genes potentially involved in human-chimpanzee dietary differences. Median sequence divergence estimates between humans and chimpanzees is shown for promoter regions (left) and for amino acid sequences (Ka/Ki) (right). The error bars represent 95% bootstrap confidence intervals for the median, calculated by resampling from the divergence estimate distributions for each gene set 1,000 times. Diet diff.-Human genes with mouse orthologs showing diet-related human-chimpanzee expression differences in liver; All diff.-Human genes with mouse orthologs showing human-chimpanzee expression differences in liver; All exprs.-Human genes with mouse orthologs expressed in liver; All genes-All available human genes with mouse orthologs.

In order to gauge the rate of evolution of the 117 genes affected by diet, we compared the DNA sequence divergence in their promoter regions [13] between humans and chimpanzees and the inferred amino acid sequences of their encoded proteins [17] to (*i*) all orthologous human and mouse genes that differ in gene expression between human and chimpanzees in liver, (*ii*) all human-mouse orthologs expressed in human or chimpanzee livers, and (*iii*) all human-mouse orthologs irrespective of their expression in liver. We find that both the promoter sequences (one-sided permutation test *p = *0.01, 0.06, 0.04, respectively) and the amino acid sequences (*p = *0.002, <0.001, 0.043, respectively) evolve faster in the 117 genes than in the latter sets of genes (Figure 3; Table S8). We also tested whether there is any significant overlap between the 117 genes that are affected by diet and differ in expression between humans and chimpanzees and either genes positively selected in their promoters in the human and chimpanzee lineages recently published by Haygood *et al.* [18] or in their amino acid sequences recently published by Bakewell *et al.* [19]. We find no such significant overlaps. This may not be surprising given the presumably high false negative rate pertaining to the identification of relevant genes in our study as well as the other studies.

## Discussion

There are several noteworthy aspects of these experiments. Regarding the general influence of diet on gene expression, it is intriguing to compare the amount of expression differences we see-4–8% of genes change between mice fed different diets for two weeks (Figure 1)-to the amount of difference observed between humans and chimpanzees: 15% [13]. Although humans and chimpanzees consume different diets their entire lives, experience many environmental differences, and have diverged genetically for more than 10 million years, the amount of gene expression differences between the two species is only about two-fold larger than that of the mice fed different diets for two weeks. Although these two numbers are not directly comparable since the mouse experiments are more controlled than the human-chimpanzee comparison and thus have more power to detect difference, the extent of expression differences induced in the liver by a change in diet is impressive.

Another interesting observation is that changes in the organism's diet have very small effects on gene expression in brain. This is not necessarily expected, given that diet has been shown to influence brain function. For example, a high fat and sugar diet affects hippocampus function in mice [20] and a polyunsaturated fatty acid diet influences genes related to synaptic plasticity and learning in the rat brain [21]. Strikingly, among the diets used in this study, only the human fast food diet had any detectable effect on gene expression in brain. This raises intriguing questions about the effects a fast food diet may have in the brain over longer times of exposure.

The two human diets differ drastically in terms of both their composition (Table S1) and their consequences for the mice in terms of weight gain (Materials and Methods). We were hence surprised to find a lack of significant differences in gene expression between the two human diets in the liver (Figure 1). This suggests that some common feature of the two human diets, which distinguishes them from both the mouse pellet and the chimpanzee diet, is responsible for these expression differences. For example, both human diets contain meat and involve cooking, features common to all or almost all human diets [2], [3], [5]. Although the common features responsible are unknown, this observation suggests that the expression changes observed in the mice represent responses to common human dietary features, rather than to the particularities of the specific diets.

The fact that genes that differ in their expression both between the mice fed human and chimpanzee diets and between humans and chimpanzees evolve faster than other genes in their promoter regions as well as their amino acid sequences suggests that changes in dietary regimes may have caused some genetic adaptations in the human and chimpanzee genomes. That dietary changes can result in genetic adaptations is illustrated, for example, by persistence of lactase expression in adults in certain human populations [9]. Alternatively, it is conceivable that certain dietary changes in human evolution, such as increased nutritional quality and a reduced need for detoxification due to the introduction of cooking, have caused a relaxation of selective constraints on diet-related genes [7]. Further work is needed to clarify this.

## Materials and Methods

### Mouse feeding regimes and array hybridization

We fed 24 female NMR1 mice one of four diets for two weeks, with six mice per diet group (Table S1). The mice were aged 8 weeks at the start of the experiment. Littermates were distributed symmetrically among groups to achieve highest possible homogeneity across groups. Water was provided *ad libidum*. The experiments described in the study were approved by the Ethics Review Committee for Animal Experimentation of the Regierungspräsidium, Leipzig. General health, behavior, and body weight of the mice were monitored throughout the study. We note that within these two weeks, the mice fed the fast food diet gained significantly more weight than the other groups (Mann-Whitney U test, *p*<0.05; Figure S2).

At the end of the 2-week period, all mice were sacrificed by cervical dislocation and liver and brain (right cerebral hemisphere) tissue were dissected. RNA was extracted from the 24 liver and brain samples as described previously [13], and processed in two batches. Both batches contained equal numbers of individuals from all diet groups. Five micrograms of RNA was used to generate labeled cRNA according to the standard Affymetrix protocol (http://www.affymetrix.com/support/technical/manual/expression_manual.affx) that was hybridized to Affymetrix® GeneChip® Mouse Genome 430 2.0 arrays. No technical replication was conducted. All expression data was deposited in NCBI Gene Expression Omnibus (http://www.ncbi.nlm.nih.gov/geo/) with accession numbers GSE6285 and GSE6297.

### Data preprocessing

Using the “affy” package from the R Bioconductor software [22], probe set expression levels (a probe set is a group of oligonucleotide probes designed to detect the expression of single gene) were calculated using the “rma” (robust multichip average) method, which includes log transformation of expression levels and quantile normalization. Detection *p*-values were calculated using the “mas5” method in the same package and only probe sets detected at *p*<0.05 in at least two individuals were included in further analyses. One mouse brain sample from the pellet diet group showed high levels of RNA degradation (data not shown), and was therefore excluded from further analysis.

### Testing for diet effects on gene expression

For each probe set we conducted a two-way ANOVA with diet and batch as factors, and compared pairs of diets using the Tukey HSD *post hoc* test (we utilized the “TukeyHSD” function in the R “stats” package). The batch in which an array was processed had a significant effect on gene expression profiles (data not shown). In order to exclude the influence of this effect on diet-related expression differences, we removed all probe sets showing a significant diet-batch interaction effect in ANOVA (at *p*<0.05) from further analyses. This is a conservative measure, but does not alter the main conclusions from the ANOVA tests (data not shown).

### Assigning Affymetrix probe sets to genes

If multiple probe sets corresponded to a single Entrez gene in the Affymetrix support table for MG-430 2.0 (http://www.affymetrix.com/support/), the minimum ANOVA *p*-value was chosen as representative. If a probe set lacked gene annotation in the Affymetrix support table, we treated it as an independent gene.

### Testing for transcriptome-wide diet effects

To assess whether the number of probe sets found to be differentially expressed among diets at ANOVA *p*<0.01 is larger than randomly expected in a data set, we used permutation tests where *(1)* the factor diet in the two-way ANOVA was randomized, *(2)* the ANOVA test was applied to all probe sets using the randomized diet factor, *(3)* the number of probe sets found to be differentially expressed at *p*<0.01 was recorded. This procedure was repeated 1,000 times. The frequency of random permutations in which the number of differentially expressed probe sets was equal to or larger than the original result was considered the *p*-value for the diet effect. In each permutation, we used the minimum *p-*value approach described above to assign *p*-values to genes with multiple probe sets. We similarly compared the results from the Tukey HSD *post hoc* test for differences between pairs of diets with the 1,000 permutations. The results are listed in Table S2.

### Testing for differences between the human and chimpanzee diets

Our results from the analyses described above indicated a significant effect of diet on mouse liver gene expression. In addition, all diet pairs showed significantly different expression levels in liver except for the cafeteria and fast food diets. We therefore combined the data from these two human diets and compared them directly to the chimpanzee diet-this approach should increase statistical power to detect gene expression differences that may be relevant to human-chimpanzee differences. For this analysis, we included only probe sets detected at *p*<0.05 in at least two individuals among the mice involved (*i.e.* the cafeteria, fast food and chimpanzee diets). We ran an ANOVA test as described above and found 830 genes (6.3% of all expressed genes) that were differentially expressed at ANOVA *p*<0.01. We then compared this result to 1,000 permutations. The permutation test *p*-value for the difference between the human and chimpanzee diets was calculated as the number of permutations in which the number of differentially expressed genes was equal to or greater than 830. We also used the median number of differentially expressed genes among the 1,000 permutations as the expected number of differentially expressed genes under the null hypothesis of no diet effect, and thus calculated an observed to expected ratio. Finally, we conducted the same analysis on pairs of other diets. Table S3 shows the results from this analysis. Note that since a large number of pairwise comparisons have been performed, the *p*-values presented in Table S3 do not represent direct measures of significance.

### Comparisons of mouse and human-chimpanzee experiments

We used previously published Affymetrix® U133plus2 gene expression data of brain and liver from six humans and five chimpanzees [13]. As with the analysis of the mouse experiment results, only probe sets detected at *p*<0.05 in at least two individuals were considered expressed. For each probe set, differential expression between the two species was calculated using a two-sided *t*-test. We calculated the proportion of differentially expressed genes using the Entrez gene annotation in the Affymetrix support table for HG-U133plus2 and the minimum *p-*value approach described above, and we chose *p*<0.01 as cut-off. For comparison with the mouse experiment, orthologous mouse and human probe sets were chosen using the Affymetrix support table. For each orthologous Entrez gene, we assigned a *p*-value for differential gene expression between the mice fed the two human diets and the chimpanzee diet, or between humans and chimpanzees by using the *p*-value from the two-way ANOVA or *t*-tests, respectively. Again we used the minimum *p-*value approach described above in assigning *p*-values to genes with multiple probe sets. The overlap between differentially expressed genes in the two experiments (at ANOVA or *t*-test *p*<0.01) was determined. Next, the *p*-value for observing such an overlap by chance given the data was calculated using 1,000 permutations generated by randomly resampling the same number of genes as were differentially expressed in the two data sets and determining the size of the overlap (Table S4). A Fisher's exact test gives qualitatively the same result: In the comparison between human and chimpanzee diet differences in mice and human and chimpanzee differences, the Fisher's exact test *p*-value for a larger than random overlap is 0.0007, while the observed to expected ratio is 1.46. The annotations of 117 human genes with mouse orthologs that were differentially expressed both between mice fed human and chimpanzee diets and between humans and chimpanzees in liver were obtained from the Affymetrix HG-U133plus2 support table and are listed in Table S5. Table S5 also contains the ANOVA and *t*-test *p*-values and effect sizes calculated using the Cohen's *d* formula (see below). Table S9 contains the ANOVA and *t*-test *p*-values and effect sizes for all 7,136 human-mouse orthologous genes expressed both in the livers of mice fed the human and chimpanzee diets and in human and chimpanzee livers.

### Effect size

Cohen's *d* has been suggested as a useful measure of the magnitude of gene expression level difference between two groups, which allows comparisons across different microarray experiments [23]. We used the following formula for effect size: *d* = (M_1_−M_2_)/SD_pooled_, where M_1_ and M_2 _are the means of the two groups and SD_pooled_ is the pooled standard deviation, calculated as SD_pooled_ = √[((N_1_−1)*SD_1_^2^)+((N_2_−1)*SD_2_^2^))/(N_1_+N_2_−2)], where N_1_ and N_2 _are the sample sizes and SD_1_ and SD_2 _are the standard deviations of the two groups. In the mouse and primate experiments, the first group was the mice fed human diets and humans, respectively. Therefore a positive (or negative) effect size indicates higher (or lower) expression in mice fed a human diet or in humans, compared to mice fed a chimpanzee diet or chimpanzees. We used the Mann-Whitney U rank test to calculate whether the absolute effect size of human-chimpanzee differences is larger in the 117 diet-related genes than all 1,169 genes differentially expressed between human and chimpanzee livers.

### Direction of expression differences

We separated the 117 diet-related genes based on positive and negative effect sizes in the mouse and primate experiments (Table S6). We then compared the correspondence between the directions in the two experiments using a Fisher's exact test.

### Gene Ontology analysis

Using the Gene Ontology (GO) [15] and a statistical tool for ontology analysis, FUNC (http://func.eva.mpg.de) [24], we tested whether the 117 diet-related genes are significantly over-represented or under-represented among GO groups. As control, we used the 1,052 human-mouse orthologs that show significant human-chimpanzee expression differences in liver but are not diet-related. 87 of the 117 genes and 711 of the 1,052 genes were found to be annotated in the GO taxonomy “Biological Process” and were used in the analysis.

The overall distribution of diet-related genes within the Biological Process taxonomy was found to be significantly non-random (FUNC taxonomy test, one-sided *p* = 0.0009). Table 1 lists the Gene Ontology categories in this taxonomy that show significant enrichment of diet-related genes after the refinement (hypergeometric test; for details about refinement see [24]).

### Expression divergence on the human and the chimpanzee lineages

We estimated the relative amounts of expression divergence on the human and the chimpanzee lineages using expression data from the livers of five orangutans measured on Affymetrix® U133plus2 arrays as an outgroup [16]. For this purpose, we calculated the log ratio between human-orangutan and chimpanzee-orangutan squared mean expression level differences for each probe set. For genes with multiple probe sets, the highest ratio was used. We then tested whether the 117 diet-related genes have greater expression divergence on the human than on the chimpanzee lineage (*i.e.* higher log ratios), relative to the three control sets of genes, using the Mann-Whitney U rank test and permutation test, as described in the previous section (Table S7).

### DNA sequence divergence

We compared DNA sequence divergence between genes potentially involved in human-chimpanzee dietary differences and other genes, using two different approaches.

First, we used the promoter divergence between human and chimpanzee in a region 1,500 base pairs upstream and 500 base pairs downstream of the transcription start site [13], and compared promoter divergence of genes affected by diet to three control sets: (1) all other human genes differently expressed between human and chimpanzee livers and having mouse orthologs (1,052 Entrez genes), (2) all other human genes expressed in human-chimpanzee livers and having mouse orthologs (7,019 Entrez genes), and (3) all other human genes having mouse orthologs (15,683 Entrez genes). For the latter analysis, the list of human-mouse orthologs was obtained by concatenating Affymetrix (http://www.affymetrix.com) and Ensembl Biomart (http://www.ensembl.org/biomart/martview/) annotation tables and including only one-to-one human-mouse orthologs.

To perform the comparisons, we used the Mann-Whitney U rank test. The significance level of the *p*-values observed in the Mann-Whitney U rank test was additionally estimated by comparing them to Mann-Whitney U rank test *p*-values calculated for 1,000 sets of the same number of genes, randomly sampled from the control sets (Table S8).

Second, we compared the levels of amino acid divergence in the genes affected by diet and in the three control sets described above. For this purpose, we used the measure of amino acid sequence divergence controlled for local substitution rates (Ka/Ki), calculated between human and chimpanzee for a list of Refseq IDs in [17]. The results of this analysis are shown in Table S8.

### Positive selection in promoter sequences on the human and chimpanzee lineages

Recently, Haygood *et al.* [18] analyzed human and chimpanzee genes for evidence for positive selection in promoter regions (5'PS). Using their results, we tested for enrichment among the 117 diet-related genes for human or chimpanzee 5'PS genes. 46 genes with the most reliable signal of positive selection in the human lineage identified by [18], with a false-discovery rate below 0.05, were matched to RefSeq mRNA IDs in the supplementary table accompanying the study [18], and these to 40 Entrez Gene IDs using the UCSC Browser “RefLink” table (http://genome.ucsc.edu/). Similarly, 62 chimpanzee 5'PS genes were matched to 56 Entrez Gene IDs. The whole set of 6,280 genes tested by [18] were also mapped to 5,590 Entrez Gene IDs.

We used the hypergeometric test to decide whether the overlap between the 117 diet-related genes and each of the 5'PS gene lists was significantly larger than expected, given the overlap between human and chimpanzee 5'PS genes and the three control sets defined above (see the Materials and Methods section “DNA sequence divergence”).

### Positive selection in amino acid sequences on the human and chimpanzee lineages

We used the results of Bakewell *et al.* [19] to test if the overlap between the 117 diet-related genes and genes positively selected in their amino acid sequences (A.A.PS) is greater than expected. There were 154 human A.A.PS genes identified in [19], which mapped to 139 Entrez genes using Ensembl Biomart (http://www.biomart.org/biomart/martview/), and 233 chimpanzee A.A.PS genes mapped to 219 Entrez genes. To test for a significant overlap, we used a table of 13,888 Ensembl Protein IDs kindly provided by Margaret Bakewell and Jianzhi Zhang, containing all genes they had tested for positive selection. We used the Ensembl Biomart to map the Ensembl Protein IDs from [19] to Ensembl Gene IDs. This did not yield a one-to-one mapping, either because some Protein ID's were out-of-date, or multiple Protein ID's matched a single Gene ID. Thus, we could map the 13,888 Ensembl Protein IDs to only 11,693 unique Entrez Gene IDs. We used the hypergeometric test to test for a significant overlap between the 117 diet-related genes and the PS gene lists, as described in the previous section.

## Supporting Information

Table S1 Mouse diet contents.
(0.03 MB DOC)

Table S2 Permutation test p-values for the number of genes affected by different diets in mice.
(0.03 MB DOC)

Table S3 Numbers of genes showing significant expression differences between mice fed different diets.
(0.06 MB DOC)

Table S4 Overlap of expression differences observed between humans and chimpanzees and between mice fed different diets.
(0.03 MB DOC)

Table S5 The 117 human-mouse orthologs showing diet-related human-chimpanzee expression differences in liver.
(0.28 MB DOC)

Table S6 Direction of expression differences among the 117 diet-related genes.
(0.03 MB DOC)

Table S7 Expression divergence on the human versus the chimpanzee lineage among the 117 diet-related genes.
(0.03 MB DOC)

Table S8 Sequence divergence patterns among the 117 diet-related genes.
(0.04 MB DOC)

Table S9 The 7,136 human-mouse orthologs expressed in human, chimpanzee and mouse livers. See Table S5 for a description of how the table contents were calculated.
(2.12 MB XLS)

Figure S1 Heatmap of expression patterns of the 117 diet-related genes. Each row represents a gene, each column a sample: Either gene expression levels in the livers of mice fed human or chimpanzee diets, or in the livers of humans or chimpanzees (from left to right). Expression values higher than the average for each gene are represented in red, values lower than the average in yellow and white.
(0.26 MB TIF)

Figure S2 Weight changes in mice fed different diets. The y-axis indicates the mean body weight among mice fed one of four different diets, measured at four days intervals during the experiment. Error bars show 95% confidence intervals of the mean based on 1,000 bootstraps. Pellet: The mouse pellet diet. Chimpanzee: The diet fed to chimpanzees in the Leipzig zoo. Cafeteria: The MPI-EVA Cafeteria diet. Fast Food: A pure McDonald's diet.
(0.27 MB TIF)
