# Supplementary material for: Human and Chimpanzee Gene Expression Differences Replicated in Mice Fed Different Diets
Source: PLoS One. 2008 Jan 30;3(1):e1504. doi: 10.1371/journal.pone.0001504 (PMC2200793; doi:10.1371/journal.pone.0001504)
Supplement: Table S1 — Mouse diet contents. (0.03 MB DOC) [file pone.0001504.s001.doc]

| **Mouse Pellet Diet** | **Chimpanzee Diet** | **Human Cafeteria Diet** | **Human Fast Food (McDonald’s™, Leipzig) Diet** |
| --- | --- | --- | --- |
| Ssniff R/M-H^TM^ pellets  16.3 MJ/Kg  58% calories from carbohydrate, 33% calories from protein, 9% calories from fat   - Dry matter (87.7%) - Raw protein (19%) - Crude fiber (4.9%) - Raw Fat (3.3%) - Ash (6.4%) - N-free extract (54.1%) - Starch (36.5%) - Sugar (4.7%) | - Bananas - Apples - Grapes - Kiwis - Pineapples - Mangos - Honeymellons - Celery - Kohlrabi - Red Beets - Porree - Stangerzeller - Cucumbers - Green bell peppers - Tomatoes - Carrots - Chicoree - Chinakohl - Lettuce - Endive - White Onions - Raisins - Plain Yogurt | - Ruccola risotto - Boiled potatoes - Sauerbraten - Red cabbage - Baked chicken breast - Stir-fried vegetables - Cooked cabbage - White rice - Grilled zucchini - Stewed tomatoes - Vegetable croquets - Fried vegetables - Sauerkraut - Breaded white fish - Gnocchi - Cous-cous - Peas - Potato-pancakes - Brusselsprouts - Roast beef - Bowtie pasta - Broccoli nut corner - Chicken wings - Tempura - Cooked corn - Spaetzle | - Cheeseburger - French fries - Apple pie - Chicken McNuggets^TM^ - Fruit & Yogurt Parfait^TM^ - Yin-Yang burger^TM^ - Filet O’Fish^TM^ - Egg McMuffin^TM^ with bacon & cheese - Big Mac^TM^ - Waffle Fries |
