# Supplementary material for: Human and Chimpanzee Gene Expression Differences Replicated in Mice Fed Different Diets
Source: PLoS One. 2008 Jan 30;3(1):e1504. doi: 10.1371/journal.pone.0001504 (PMC2200793; doi:10.1371/journal.pone.0001504)
Supplement: Table S2 — Permutation test p-values for the number of genes affected by different diets in mice. (0.03 MB DOC) [file pone.0001504.s002.doc]

|  | | **Permutation test *p*-values ^a^** | | | | | | |
| --- | --- | --- | --- | --- | --- | --- | --- | --- |
| **Tissue** | **Number of PS**  **^b^** | **All**  **Diets^c^** | **Cafe-**  **F.Food^d^** | **Cafe-**  **Chimp^d^** | **F.Food-**  **Chimp^d^** | **Pellet-**  **Cafe^d^** | **Pellet-**  **F.Food^d^** | **Pellet-**  **Chimp^d^** |
| **Liver** | 13557 | 0.004 | 0.151 | 0.019 | 0.017 | 0.004 | 0.004 | 0.001 |
| **Brain** | 7523 | 0.156 | 0.230 | 0.197 | 0.142 | 0.311 | 0.032 | 0.110 |

**^a^** The *p*-value for the number of differentially expressed genes (at ANOVA or Tukey’s HSD test *p* < 0.01) between mice fed different diets being larger than random, based on 1,000 permutations. See Materials and Methods for details.

**^b^** Number of expressed probe sets. Probe sets showing significant interaction effects (at ANOVA *p*<0.05 for the batch-diet interaction effect) are excluded, and a single probe set is chosen per Entrez gene using the minimum *p-*value approach described in Materials and Methods.

**^c^** Permutation test *p*-value for the number of differentially expressed genes among all four diets (at *p* < 0.01, ANOVA).

**^d^** Permutation test *p*-value for the number of differentially expressed genes between two diets (at *p* < 0.01, Tukey’s HSD). Diets are labeled as follows: **Cafe** -human cafeteria diet; **F.Food** – human fast food diet; **Chimp** -chimpanzee diet; **Pellet** - mouse pellet diet.
