# Supplementary material for: Human and Chimpanzee Gene Expression Differences Replicated in Mice Fed Different Diets
Source: PLoS One. 2008 Jan 30;3(1):e1504. doi: 10.1371/journal.pone.0001504 (PMC2200793; doi:10.1371/journal.pone.0001504)
Supplement: Table S3 — Numbers of genes showing significant expression differences between mice fed different diets. (0.06 MB DOC) [file pone.0001504.s003.doc]

| **Tissue** | **Diet 1** | **Diet 2** | **# Genes**  **expressed ^a^** | **# Genes**  **changed**  **^b^** | **% Genes**  **changed** | **Permutation**  **test *p*-value^c^** | **Observed/**  **expected^d^** |
| --- | --- | --- | --- | --- | --- | --- | --- |
| **Liver** | Chimp | Cafe+F.Food | 13168 | 830 | 6.3 | 0.030 | 7.094 |
| **Liver** | Pellet | Cafe+F.Food | 13286 | 1184 | 8.9 | 0.010 | 9.626 |
| **Liver** | F.Food | Cafe+Chimp | 13168 | 387 | 2.9 | 0.115 | 2.932 |
| **Liver** | Cafe | F.Food | 12265 | 125 | 1.0 | 0.397 | 1.269 |
| **Liver** | Chimp | Cafe | 12064 | 502 | 4.2 | 0.103 | 4.648 |
| **Liver** | Chimp | F.Food | 12357 | 993 | 8.0 | 0.030 | 8.139 |
| **Liver** | Pellet | Chimp | 12358 | 2263 | 18.3 | 0.003 | 17.680 |
| **Liver** | Pellet | Cafe | 12259 | 853 | 7.0 | 0.010 | 7.353 |
| **Liver** | Pellet | F.Food | 12529 | 1013 | 8.1 | 0.015 | 7.853 |
| **Brain** | Chimp | Cafe+F.Food | 16753 | 325 | 1.9 | 0.191 | 1.890 |
| **Brain** | F.Food | Cafe+Chimp | 16753 | 530 | 3.2 | 0.104 | 2.880 |
| **Brain** | Cafe | F.Food | 16048 | 383 | 2.4 | 0.132 | 2.266 |
| **Brain** | Chimp | Cafe | 15965 | 191 | 1.2 | 0.411 | 1.172 |
| **Brain** | Chimp | F.Food | 16032 | 364 | 2.3 | 0.150 | 2.233 |
| **Brain** | Pellet | Chimp | 11649 | 152 | 1.3 | 0.250 | 1.551 |
| **Brain** | Pellet | Cafe | 11676 | 144 | 1.2 | 0.387 | 1.220 |
| **Brain** | Pellet | F.Food | 11835 | 424 | 3.6 | 0.064 | 3.786 |

**^a^** Genes with detectable expression in at least two individuals among the groups of mice fed the relevant diets.

**^b^** Genes differentially expressed between mice fed the two diets (*p* < 0.01, ANOVA).

**^c^** The *p*-value for transcriptome-wide diet effects calculated as described in Materials and Methods.

**^d^** The ratio between observed number of changed genes and median number of changed genes in 1,000 permutations.

Diets are labeled as follows: **Pellet** - mouse pellet diet; **Chimp** -chimpanzee diet; **Cafe** -human cafeteria diet; **F.Food** – human fast food diet; **Cafe+F.Food** - human cafeteria and fast food diets together; **Cafe+Chimp** - human cafeteria and chimpanzee diets together.
