# Supplementary material for: Human and Chimpanzee Gene Expression Differences Replicated in Mice Fed Different Diets
Source: PLoS One. 2008 Jan 30;3(1):e1504. doi: 10.1371/journal.pone.0001504 (PMC2200793; doi:10.1371/journal.pone.0001504)
Supplement: Table S4 — Overlap of expression differences observed between humans and chimpanzees and between mice fed different diets. (0.03 MB DOC) [file pone.0001504.s004.doc]

| **Experiments compared** | | **# Genes expressed in both ^c^** | **# Genes changed in both ^d^** | **Permutation test *p*-value ^e^** | **Observed/**  **expected ratio ^f^** |
| --- | --- | --- | --- | --- | --- |
| **Mouse ^a^** | **Human-Chimpanzee ^b^** |
| **Liver H-C** | **Liver** | 7136 | 117 | 0.001 | 1.330 |
| **Liver H-P** | **Liver** | 7226 | 124 | 0.673 | 0.973 |
| **Liver H-C** | **Brain** | 6981 | 122 | 0.417 | 1.017 |
| **Brain H-C** | **Liver** | 7788 | 25 | 0.685 | 0.926 |
| **Brain H-C** | **Brain** | 8290 | 45 | 0.548 | 1.000 |

**^a^** Genes differentially expressed between mice fed human and chimpanzee diets (H-C) or mice fed human and mouse pellet diets (H-P), in either liver or brain.

**^b^** Genes differentially expressed between humans and chimpanzees, in either liver or brain.

**^c^** The number of genes with detectable expression both in the mouse experiment and in humans and chimpanzees.

**^d^** The number of genes differentially expressed in both experiments (at ANOVA or t-test *p*<0.01).

**^e^** The *p*-value based on 1,000 permutations (see Materials and Methods).

**^f^** The ratio between the observed number of genes differentially expressed in both experiments and the median number of such genes in 1,000 permutations.
