# Supplementary material for: Human and Chimpanzee Gene Expression Differences Replicated in Mice Fed Different Diets
Source: PLoS One. 2008 Jan 30;3(1):e1504. doi: 10.1371/journal.pone.0001504 (PMC2200793; doi:10.1371/journal.pone.0001504)
Supplement: Table S5 — The 117 human-mouse orthologs showing diet-related human-chimpanzee expression differences in liver. (0.28 MB DOC) [file pone.0001504.s005.doc]

| **Entrez Gene ID** | **Gene Title** | **Gene Symbol** | **Ensembl Gene ID** | **RefSeq Transcript ID** | **Affymetrix MG430 2.0 Probe Set** | **Effect size – mouse^a^** | ***P*-value – mouse^b^** | **Affymetrix HG U133Plus2 Probe Set** | **Effect size - human-chimpanzee^c^** | ***P*-value - human-chimpanzee^d^** |
| --- | --- | --- | --- | --- | --- | --- | --- | --- | --- | --- |
| 18 | 4-aminobutyrate aminotransferase | ABAT | ENSG00000183044 | NM_000663 /// NM_020686 | 1433855_at | 2.47 | 2.74E-04 | 209460_at | 5.10 | 7.59E-04 |
| 127 | Alcohol dehydrogenase 4 (class II), pi polypeptide | ADH4 | ENSG00000198099 | NM_000670 | 1422070_at | 1.87 | 3.26E-03 | 231703_s_at | 5.27 | 1.08E-04 |
| 189 | Alanine-glyoxylate aminotransferase (oxalosis I; hyperoxaluria I; glycolicaciduria; serine-pyruvate aminotransferase) | AGXT | ENSG00000172482 | NM_000030 | 1418833_at | 2.81 | 1.14E-04 | 206957_at | 6.84 | 1.33E-06 |
| 219 | Aldehyde dehydrogenase 1 family, member B1 | ALDH1B1 | ENSG00000137124 | NM_000692 | 1451260_at | 1.75 | 5.52E-03 | 209646_x_at | 2.51 | 6.02E-03 |
| 308 | Annexin A5 | ANXA5 | ENSG00000164111 | NM_001154 | 1425567_a_at | 2.12 | 1.31E-03 | 200782_at | -2.67 | 2.34E-03 |
| 839 | Caspase 6, apoptosis-related cysteine protease | CASP6 | ENSG00000138794 | NM_001226 /// NM_032992 | 1415995_at | 2.22 | 9.04E-04 | 211464_x_at | -5.92 | 1.66E-04 |
| 875 | Cystathionine-beta-synthase | CBS | ENSG00000160200 | NM_000071 | 1425623_a_at | 2.27 | 8.24E-04 | 240517_at | -2.80 | 1.08E-03 |
| 966 | CD59 antigen p18-20 (antigen identified by monoclonal antibodies 16.3A5, EJ16, EJ30, EL32 and G344) | CD59 | ENSG00000085063 | NM_000611 /// NM_203329 /// NM_203330 /// NM_203331 | 1429830_a_at | 2.31 | 6.94E-04 | 200984_s_at | 3.04 | 2.61E-03 |
| 977 | CD151 antigen | CD151 | ENSG00000177697 | NM_004357 /// NM_139030 | 1456085_x_at | 1.61 | 6.70E-03 | 204306_s_at | 4.05 | 1.32E-04 |
| 1355 | COX15 homolog, cytochrome c oxidase assembly protein (yeast) | COX15 | ENSG00000014919 | NM_004376 /// NM_078470 | 1426693_x_at | 1.87 | 2.98E-03 | 223281_s_at | -3.73 | 2.00E-03 |
| 1586 | Cytochrome P450, family 17, subfamily A, polypeptide 1 | CYP17A1 | ENSG00000148795 | NM_000102 | 1417017_at | -2.00 | 1.96E-03 | 205502_at | -3.44 | 3.53E-03 |
| 1622 | Diazepam binding inhibitor (GABA receptor modulator, acyl-Coenzyme A binding protein) | DBI | ENSG00000155368 | NM_020548 | 1422432_at | 1.66 | 7.35E-03 | 209389_x_at | 2.55 | 8.88E-03 |
| 1717 | 7-dehydrocholesterol reductase | DHCR7 | ENSG00000172893 | NM_001360 | 1448619_at | 1.85 | 1.66E-03 | 201791_s_at | 2.29 | 3.75E-03 |
| 1827 | Down syndrome critical region gene 1 | DSCR1 | ENSG00000159200 | NM_004414 /// NM_203417 /// NM_203418 | 1416600_a_at | -2.37 | 2.86E-04 | 208370_s_at | 3.54 | 4.49E-04 |
| 2230 | Ferredoxin 1 | FDX1 | ENSG00000137714 | NM_004109 | 1449108_at | 1.64 | 7.90E-03 | 239161_at | 3.49 | 2.40E-04 |
| 2246 | Fibroblast growth factor 1 (acidic) | FGF1 | ENSG00000113578 | NM_000800 /// NM_033136 /// NM_033137 | 1423136_at | -1.71 | 6.18E-03 | 208240_s_at | -2.45 | 3.05E-03 |
| 2335 | Fibronectin 1 | FN1 | ENSG00000115414 | NM_002026 /// NM_054034 /// NM_212474 /// NM_212475 /// NM_212476 /// NM_212478 /// NM_212482 | 1426642_at | -1.79 | 4.57E-03 | 212464_s_at | 3.98 | 1.60E-03 |
| 2628 | Glycine amidinotransferase (L-arginine:glycine amidinotransferase) | GATM | ENSG00000171766 | NM_001482 | 1423569_at | 1.54 | 7.52E-03 | 231686_at | 2.86 | 1.41E-03 |
| 2752 | Glutamate-ammonia ligase (glutamine synthase) | GLUL | ENSG00000135821 | NM_002065 | 1426235_a_at | -1.78 | 4.55E-03 | 242281_at | 2.67 | 2.95E-03 |
| 2953 | Glutathione S-transferase theta 2 | GSTT2 | ENSG00000133433 | NM_000854 | 1417883_at | 1.67 | 7.15E-03 | 205439_at | -4.22 | 1.39E-04 |
| 2982 | Guanylate cyclase 1, soluble, alpha 3 | GUCY1A3 | ENSG00000164116 | NM_000856 | 1434141_at | 1.96 | 2.26E-03 | 221942_s_at | -2.15 | 5.88E-03 |
| 3176 | Histamine N-methyltransferase | HNMT | ENSG00000150540 | NM_006895 | 1417702_a_at | 1.54 | 9.74E-03 | 228772_at | 2.43 | 2.60E-03 |
| 3554 | Interleukin 1 receptor, type I | IL1R1 | ENSG00000115594 | NM_000877 | 1448950_at | -1.93 | 1.94E-03 | 202948_at | -3.26 | 2.34E-03 |
| 3625 | Inhibin, beta B (activin AB beta polypeptide) | INHBB | ENSG00000163083 | NM_002193 | 1426858_at | -3.41 | 5.70E-06 | 205258_at | 2.40 | 4.57E-03 |
| 3712 | Isovaleryl Coenzyme A dehydrogenase | IVD | ENSG00000128928 | NM_002225 | 1449001_at | 3.80 | 1.70E-06 | 225311_at | 3.98 | 8.16E-04 |
| 3960 | Lectin, galactoside-binding, soluble, 4 (galectin 4) | LGALS4 | ENSG00000171747 | NM_006149 | 1451336_at | 1.67 | 4.56E-03 | 204272_at | 3.85 | 5.34E-04 |
| 4013 | Loss of heterozygosity, 11, chromosomal region 2, gene A | LOH11CR2A | ENSG00000110002 | NM_014622 /// NM_198315 | 1426221_at | 2.39 | 4.50E-04 | 205011_at | -3.26 | 1.03E-03 |
| 4077 | Neighbor of BRCA1 gene 1 | M17S2 | ENSG00000196191 | NM_005899 /// NM_031858 /// NM_031862 | 1451897_a_at | 1.99 | 1.04E-03 | 1568857_a_at | -3.52 | 1.55E-03 |
| 4522 | Methylenetetrahydrofolate dehydrogenase (NADP+ dependent) 1, methenyltetrahydrofolate cyclohydrolase, formyltetrahydrofolate synthetase | MTHFD1 | ENSG00000100714 | NM_005956 | 1415917_at | 2.06 | 1.68E-03 | 202309_at | -2.05 | 6.89E-03 |
| 4704 | NADH dehydrogenase (ubiquinone) 1 alpha subcomplex, 9, 39kda | NDUFA9 | ENSG00000139180 | NM_005002 | 1416663_at | 1.61 | 4.02E-03 | 208969_at | -3.68 | 2.27E-04 |
| 4718 | NADH dehydrogenase (ubiquinone) 1, subcomplex unknown, 2, 14.5kda | NDUFC2 | ENSG00000151366 | NM_004549 | 1460506_s_at | 1.89 | 2.17E-03 | 222521_x_at | 2.66 | 6.42E-03 |
| 5002 | Solute carrier family 22 (organic cation transporter), member 18 | SLC22A18 | ENSG00000110628 | NM_002555 /// NM_183233 | 1417809_at | 1.68 | 3.92E-03 | 204981_at | -2.08 | 6.45E-03 |
| 5096 | Propionyl Coenzyme A carboxylase, beta polypeptide | PCCB | ENSG00000114054 | NM_000532 | 1450969_at | 1.62 | 8.86E-03 | 212694_s_at | 4.09 | 1.75E-04 |
| 5169 | Ectonucleotide pyrophosphatase/phosphodiesterase 3 | ENPP3 | ENSG00000154269 | NM_005021 | 1452384_at | 1.84 | 2.06E-03 | 232737_s_at | 4.04 | 7.71E-05 |
| 5514 | Protein phosphatase 1, regulatory subunit 10 | PPP1R10 | ENSG00000137314 | NM_002714 | 1430560_at | 1.68 | 7.09E-03 | 201703_s_at | 3.31 | 3.36E-04 |
| 5959 | Retinol dehydrogenase 5 (11-cis and 9-cis) | RDH5 | ENSG00000135437 | NM_002905 | 1418808_at | 2.03 | 1.83E-03 | 210106_at | -2.49 | 6.36E-03 |
| 6500 | S-phase kinase-associated protein 1A (p19a) | SKP1A | ENSG00000113558 | NM_006930 /// NM_170679 | 1423149_at | 1.56 | 7.73E-03 | 200711_s_at | 3.08 | 1.62E-03 |
| 6540 | Solute carrier family 6 (neurotransmitter transporter, GABA), member 13 | SLC6A13 | ENSG00000010379 | NM_016615 | 1424338_at | -2.18 | 1.11E-03 | 207184_at | 2.47 | 2.52E-03 |
| 6542 | Solute carrier family 7 (cationic amino acid transporter, y+ system), member 2 | SLC7A2 | ENSG00000003989 | NM_001008539 /// NM_003046 | 1426008_a_at | 1.81 | 4.17E-03 | 225516_at | 2.35 | 3.68E-03 |
| 6568 | Solute carrier family 17 (sodium phosphate), member 1 | SLC17A1 | ENSG00000124568 | NM_005074 | 1417280_at | -1.93 | 2.48E-03 | 237049_at | 2.63 | 3.60E-03 |
| 6902 | Tubulin-specific chaperone a | TBCA | ENSG00000171530 | NM_004607 | 1434588_x_at | 1.59 | 8.04E-03 | 203667_at | 9.64 | 1.57E-07 |
| 7172 | Thiopurine S-methyltransferase | TPMT | ENSG00000137364 | NM_000367 | 1430889_a_at | 2.21 | 3.37E-04 | 203672_x_at | 3.62 | 1.52E-03 |
| 7320 | Ubiquitin-conjugating enzyme E2B (RAD6 homolog) | UBE2B | ENSG00000119048 | NM_003337 | 1423107_at | 1.88 | 3.16E-03 | 228588_s_at | 2.11 | 6.41E-03 |
| 7726 | Tripartite motif-containing 26 | TRIM26 | ENSG00000137313 | NM_003449 | 1424929_a_at | 1.85 | 3.76E-03 | 235788_at | -2.99 | 8.57E-03 |
| 8869 | ST3 beta-galactoside alpha-2,3-sialyltransferase 5 | ST3GAL5 | ENSG00000115525 | NM_003896 | 1449198_a_at | -1.64 | 7.60E-03 | 203217_s_at | -2.41 | 3.30E-03 |
| 9050 | Proline-serine-threonine phosphatase interacting protein 2 | PSTPIP2 | ENSG00000152229 | NM_024430 | 1421410_a_at | 1.70 | 5.87E-03 | 219938_s_at | -3.72 | 4.29E-03 |
| 9060 | 3'-phosphoadenosine 5'-phosphosulfate synthase 2 | PAPSS2 | ENSG00000198682 | NM_001015880 /// NM_004670 | 1421987_at | 3.16 | 3.52E-05 | 203059_s_at | -3.03 | 1.88E-03 |
| 9563 | Hexose-6-phosphate dehydrogenase (glucose 1-dehydrogenase) | H6PD | ENSG00000049239 | NM_004285 | 1452145_at | -2.10 | 1.48E-03 | 221892_at | 2.52 | 2.09E-03 |
| 9921 | Ring finger protein 10 | RNF10 | ENSG00000022840 | NM_014868 | 1423102_a_at | -1.61 | 9.26E-03 | 237062_at | 2.49 | 2.21E-03 |
| 10079 | Atpase, Class II, type 9A | ATP9A | ENSG00000054793 | XM_030577 | 1427604_a_at | 1.64 | 8.40E-03 | 212062_at | 1.98 | 9.19E-03 |
| 10140 | Transducer of ERBB2, 1 | TOB1 | ENSG00000141232 | NM_005749 | 1423176_at | 2.81 | 1.11E-04 | 228834_at | 3.44 | 3.09E-04 |
| 10186 | Lipoma HMGIC fusion partner | LHFP | ENSG00000183722 | NM_005780 | 1433776_at | 2.05 | 1.02E-03 | 231411_at | -2.33 | 9.17E-03 |
| 10240 | Mitochondrial ribosomal protein S31 | MRPS31 | ENSG00000102738 | NM_005830 | 1417737_at | 1.65 | 8.05E-03 | 212604_at | -2.36 | 3.13E-03 |
| 10425 | Ariadne homolog 2 (Drosophila) | ARIH2 | ENSG00000177479 | NM_006321 | 1418523_at | -1.84 | 1.78E-03 | 201229_s_at | -2.40 | 2.94E-03 |
| 10645 | Calcium/calmodulin-dependent protein kinase kinase 2, beta | CAMKK2 | ENSG00000110931 | NM_006549 /// NM_153499 /// NM_153500 /// NM_172214 /// NM_172215 /// NM_172216 /// NM_172226 | 1455401_at | 1.50 | 7.78E-03 | 212252_at | 3.83 | 4.81E-04 |
| 10667 | Phenylalanine-trna synthetase 2 (mitochondrial) | FARS2 | ENSG00000145982 | NM_006567 | 1431354_a_at | 1.85 | 2.59E-03 | 204283_at | -2.56 | 4.80E-03 |
| 11185 | Indolethylamine N-methyltransferase | INMT | ENSG00000011177 | NM_006774 | 1418697_at | 2.65 | 1.69E-04 | 224061_at | -2.56 | 3.67E-03 |
| 22868 | Kiaa0971 | KIAA0971 | ENSG00000118246 | NM_014929 | 1452172_at | 1.65 | 5.52E-03 | 205976_at | -3.11 | 8.88E-03 |
| 22926 | Activating transcription factor 6 | ATF6 | ENSG00000118217 | NM_007348 | 1435444_at | -1.95 | 2.03E-03 | 239825_at | 3.54 | 5.14E-04 |
| 23327 | Neural precursor cell expressed, developmentally down-regulated 4-like | NEDD4L | ENSG00000049759 | NM_015277 | 1441305_at | 1.64 | 8.35E-03 | 212445_s_at | -2.69 | 5.04E-03 |
| 23424 | Tudor domain containing 7 | TDRD7 | ENSG00000196116 | NM_014290 | 1426716_at | 1.81 | 4.10E-03 | 213361_at | -2.16 | 5.91E-03 |
| 23475 | Quinolinate phosphoribosyltransferase (nicotinate-nucleotide pyrophosphorylase (carboxylating)) | QPRT | ENSG00000103485 | NM_014298 | 1418836_at | 2.59 | 1.03E-04 | 242414_at | 4.96 | 2.51E-05 |
| 23576 | Dimethylarginine dimethylaminohydrolase 1 | DDAH1 | ENSG00000153904 | NM_012137 | 1429298_at | 2.13 | 8.91E-04 | 229456_s_at | 2.44 | 2.55E-03 |
| 23589 | Calcium regulated heat stable protein 1, 24kda | CARHSP1 | ENSG00000153048 | NM_014316 | 1415976_a_at | 1.61 | 4.80E-03 | 224910_at | 2.67 | 2.80E-03 |
| 25950 | RWD domain containing 3 | RWDD3 | ENSG00000122481 | NM_015485 | 1430167_a_at | 1.62 | 8.37E-03 | 205087_at | 4.28 | 6.79E-05 |
| 26012 | Nasal embryonic LHRH factor | NELF | ENSG00000165802 | NM_015537 | 1449474_a_at | -1.71 | 5.41E-03 | 221214_s_at | -2.33 | 3.36E-03 |
| 26118 | WD repeat and SOCS box-containing 1 | WSB1 | ENSG00000109046 | NM_015626 /// NM_134264 /// NM_134265 | 1425241_a_at | 2.45 | 2.98E-04 | 201295_s_at | -2.34 | 7.00E-03 |
| 26286 | ADP-ribosylation factor gtpase activating protein 3 | ARFGAP3 | ENSG00000100262 | NM_014570 | 1426534_a_at | -1.62 | 4.93E-03 | 202211_at | -2.35 | 4.82E-03 |
| 28957 | Mitochondrial ribosomal protein S28 | MRPS28 | ENSG00000147586 | NM_014018 | 1452585_at | 1.85 | 1.83E-03 | 236955_at | 1.99 | 9.14E-03 |
| 51065 | Ribosomal protein S27-like | RPS27L | ENSG00000185088 | NM_015920 | 1423254_x_at | 1.75 | 4.62E-03 | 238935_at | 5.51 | 1.01E-05 |
| 51166 | Aminoadipate aminotransferase | AADAT | ENSG00000109576 | NM_016228 /// NM_182662 | 1418519_at | 2.32 | 2.63E-04 | 223593_at | 5.86 | 6.65E-05 |
| 51179 | Hydroxyacid oxidase 2 (long chain) | HAO2 | ENSG00000116882 | NM_001005783 /// NM_016527 | 1418654_at | 3.39 | 1.39E-05 | 231156_at | 7.43 | 1.98E-06 |
| 51181 | Dicarbonyl/L-xylulose reductase | DCXR | ENSG00000169738 | NM_016286 | 1419456_at | 2.18 | 6.06E-04 | 217973_at | 3.36 | 4.69E-04 |
| 51226 | Coatomer protein complex, subunit zeta 2 | COPZ2 | ENSG00000005243 | NM_016429 | 1418455_at | 3.33 | 2.18E-05 | 219561_at | -3.65 | 4.51E-04 |
| 51263 | Mitochondrial ribosomal protein L30 | MRPL30 | ENSG00000185414 | NM_016503 /// NM_145212 /// NM_145213 | 1423857_at | 1.59 | 8.40E-03 | 243887_at | 2.28 | 8.01E-03 |
| 51302 | Cytochrome P450, family 39, subfamily A, polypeptide 1 | CYP39A1 | ENSG00000146233 | NM_016593 | 1418780_at | -2.36 | 3.85E-04 | 244407_at | 2.73 | 1.52E-03 |
| 51380 | Cysteine sulfinic acid decarboxylase | CSAD | ENSG00000139631 | NM_015989 | 1427981_a_at | -1.60 | 6.61E-03 | 1568620_at | 4.36 | 2.38E-04 |
| 51390 | Androgen-induced 1 | AIG1 | ENSG00000146416 | NM_016108 | 1420679_a_at | 1.80 | 3.78E-03 | 1556211_a_at | 3.66 | 2.50E-04 |
| 51409 | Hemk methyltransferase family member 1 | HEMK1 | ENSG00000114735 | NM_016173 | 1424703_at | 1.64 | 8.44E-03 | 52159_at | 3.48 | 2.68E-04 |
| 51776 | Sterile alpha motif and leucine zipper containing kinase AZK | ZAK | ENSG00000091436 | NM_016653 /// NM_133646 | 1418943_at | 1.58 | 9.25E-03 | 225665_at | -2.77 | 1.27E-03 |
| 54494 | Hypothetical protein FLJ20010 | FLJ20010 | ENSG00000180425 | NM_019021 | 1423889_at | 2.81 | 4.56E-05 | 218789_s_at | 4.32 | 8.45E-05 |
| 54838 | Chromosome 10 open reading frame 26 | C10orf26 | ENSG00000166272 | NM_017787 | 1433717_at | -2.03 | 1.88E-03 | 202808_at | -4.16 | 2.05E-03 |
| 54902 | Tetratricopeptide repeat domain 19 | TTC19 | ENSG00000011295 | NM_017775 | 1427114_at | 1.86 | 3.58E-03 | 217964_at | 2.51 | 5.76E-03 |
| 54962 | Timeless-interacting protein | FLJ20516 | ENSG00000075131 | NM_017858 | 1426612_at | 1.67 | 7.56E-03 | 219258_at | 2.63 | 1.67E-03 |
| 55093 | Hypothetical protein FLJ10204 | FLJ10204 | ENSG00000156795 | NM_018024 | 1432489_a_at | 1.95 | 9.01E-04 | 219060_at | -6.11 | 1.11E-04 |
| 55825 | Peroxisomal trans-2-enoyl-coa reductase | PECR | ENSG00000115425 | NM_018441 | 1448910_at | 2.12 | 7.34E-04 | 221142_s_at | -3.77 | 1.51E-04 |
| 56888 | Potassium channel modulatory factor 1 | KCMF1 | ENSG00000176407 | NM_020122 | 1449180_at | -2.02 | 1.93E-03 | 242887_at | 4.69 | 6.60E-04 |
| 58510 | Proline dehydrogenase (oxidase) 2 | PRODH2 | ENSG00000161270 | NM_021232 | 1432099_a_at | 1.78 | 4.60E-03 | 216696_s_at | 4.34 | 2.12E-03 |
| 64087 | Methylcrotonoyl-Coenzyme A carboxylase 2 (beta) | MCCC2 | ENSG00000131844 | NM_022132 | 1428021_at | 2.80 | 7.25E-05 | 209623_at | 3.56 | 7.53E-04 |
| 64343 | 5-azacytidine induced 2 | AZI2 | ENSG00000163512 | NM_022461 /// NM_203326 | 1448032_at | -1.80 | 4.15E-03 | 227904_at | 2.28 | 3.86E-03 |
| 64432 | Mitochondrial ribosomal protein S25 | MRPS25 | ENSG00000131368 | NM_022497 | 1449194_at | 1.59 | 9.99E-03 | 224873_s_at | 3.42 | 1.97E-03 |
| 64776 | Chromosome 11 open reading frame 1 | C11orf1 | ENSG00000137720 | NM_022761 | 1417211_a_at | 2.18 | 1.13E-03 | 231530_s_at | 3.95 | 8.81E-04 |
| 79622 | Chromosome 16 open reading frame 33 | C16orf33 | ENSG00000161981 | NM_024571 | 1428004_at | 2.93 | 4.83E-05 | 218493_at | -2.35 | 7.22E-03 |
| 79641 | Leucine zipper domain protein | FLJ22386 | ENSG00000067836 | NM_024589 | 1451421_a_at | 1.91 | 2.67E-03 | 218394_at | -4.07 | 1.47E-03 |
| 79689 | Tumor necrosis factor, alpha-induced protein 9 | TNFAIP9 | ENSG00000127954 | NM_024636 | 1425829_a_at | 1.74 | 5.72E-03 | 225987_at | -3.13 | 2.43E-03 |
| 79746 | Enoyl Coenzyme A hydratase domain containing 3 | ECHDC3 | ENSG00000134463 | NM_024693 | 1418862_at | 2.93 | 7.16E-05 | 219298_at | -2.49 | 2.26E-03 |
| 79783 | Chromosome 7 open reading frame 10 | C7orf10 | ENSG00000175600 | NM_024728 | 1421422_at | 2.06 | 1.56E-03 | 219655_at | 2.25 | 4.11E-03 |
| 79784 | Myosin, heavy polypeptide 14 | MYH14 | ENSG00000105357 | NM_024729 | 1428835_at | 2.23 | 5.06E-04 | 232977_x_at | -3.68 | 4.42E-04 |
| 79791 | F-box protein 31 | FBXO31 | ENSG00000103264 | NM_024735 | 1417969_at | -2.06 | 1.47E-03 | 219785_s_at | -3.48 | 8.25E-04 |
| 79984 | Esterase 31 | FLJ21736 | ENSG00000172828 | NM_024922 | 1451600_s_at | 1.99 | 1.08E-03 | 220335_x_at | 4.19 | 1.89E-04 |
| 80017 | Chromosome 14 open reading frame 159 | C14orf159 | ENSG00000133943 | NM_024952 | 1424226_at | 1.66 | 7.72E-03 | 218298_s_at | -5.72 | 6.09E-06 |
| 80095 | Zinc finger protein 606 | ZNF606 | ENSG00000166704 | NM_025027 | 1436429_at | -1.70 | 4.92E-03 | 229707_at | -2.04 | 7.18E-03 |
| 80194 | Hypothetical protein FLJ21749 | FLJ21749 | ENSG00000172663 | NM_025124 | 1455752_a_at | 1.95 | 2.08E-03 | 218531_at | -2.78 | 1.11E-03 |
| 83988 | Neurocalcin delta /// neurocalcin delta | NCALD | ENSG00000104490 | NM_032041 | 1417569_at | 2.01 | 2.10E-03 | 211685_s_at | 2.76 | 3.44E-03 |
| 84105 | 6-pyruvoyl-tetrahydropterin synthase/dimerization cofactor of hepatocyte nuclear factor 1 alpha (TCF1) 2 | PCBD2 | ENSG00000132570 | NM_032151 | 1452621_at | 1.69 | 4.22E-03 | 223712_at | -4.27 | 6.76E-05 |
| 85007 | Hypothetical protein MGC15875 | MGC15875 | ENSG00000175309 | NM_032921 /// NM_153373 | 1424745_at | 1.97 | 2.41E-03 | 232488_at | -2.11 | 5.91E-03 |
| 85458 | DIX domain containing 1 | DIXDC1 | ENSG00000150764 | NM_033425 | 1435207_at | 2.03 | 1.49E-03 | 214724_at | 2.23 | 4.51E-03 |
| 91612 | Churchill domain containing 1 | CHURC1 | ENSG00000197195 | NM_145165 | 1425455_a_at | 1.77 | 3.55E-03 | 226736_at | -2.62 | 2.07E-03 |
| 92105 | Hypothetical gene MGC16733 similar to CG12113 | MGC16733 | ENSG00000149262 | NM_033547 | 1423806_at | 1.92 | 1.97E-03 | 1553670_at | -3.48 | 2.37E-03 |
| 93974 | ATPase inhibitory factor 1 | ATPIF1 | ENSG00000130770 | NM_016311 /// NM_178190 /// NM_178191 | 1448770_a_at | 2.40 | 5.09E-04 | 223339_at | 2.54 | 4.22E-03 |
| 123355 | Leucine rich repeat containing 28 | LRRC28 | ENSG00000168904 | NM_144598 | 1433858_at | -2.42 | 4.67E-04 | 1553940_a_at | 2.22 | 8.35E-03 |
| 130502 | Similar to CG14894-PA | LOC130502 | ENSG00000183891 | NM_001008237 | 1452972_at | 1.70 | 5.96E-03 | 226838_at | -2.03 | 8.71E-03 |
| 150737 | Hypothetical protein FLJ30990 | FLJ30990 | ENSG00000197557 | NM_152517 | 1423672_at | 1.82 | 3.95E-03 | 1554588_a_at | -2.83 | 6.47E-03 |
| 158234 | RNA (guanine-9-) methyltransferase domain containing 3 | RG9MTD3 | ENSG00000165275 | NM_144964 | 1426494_at | 1.54 | 9.64E-03 | 235303_at | -4.01 | 4.66E-04 |
| 203197 | Chromosome 9 open reading frame 91 | C9orf91 | ENSG00000157693 | NM_153045 | 1426316_at | -1.69 | 5.32E-03 | 221865_at | -4.39 | 5.37E-05 |
| 284106 | Hypothetical protein LOC284106 | LOC284106 | ENSG00000188260 | XM_375449 | 1435017_at | 2.18 | 1.04E-03 | 226727_at | 2.11 | 9.15E-03 |
| 285343 | Hypothetical protein dkfzp313n0621 | DKFZp313N0621 | ENSG00000179152 | NM_173826 | 1455118_at | 1.57 | 9.88E-03 | 226688_at | 2.30 | 4.57E-03 |

Gene identifiers in columns 1-5 are obtained from the Affymetrix support site (www.affymetrix.com).

**^a^** The effect size (Cohen’s *d*; see Materials and Methods) comparing gene expression levels in livers of mice fed human diets and the chimpanzee diet (positive values indicate higher expression in mice fed the human diets).

**^b^** The *p*-value from the ANOVA test for a difference between gene expression levels in livers of mice fed the two human diets and the chimpanzee diet.

**^c^** The effect size (Cohen’s *d*) comparing gene expression levels in livers of humans and chimpanzees (positive values indicate higher expression in humans).

**^d^** The *p*-value from the *t*-test for a difference between gene expression levels in livers of humans and chimpanzees.
