# Supplementary material for: Human and Chimpanzee Gene Expression Differences Replicated in Mice Fed Different Diets
Source: PLoS One. 2008 Jan 30;3(1):e1504. doi: 10.1371/journal.pone.0001504 (PMC2200793; doi:10.1371/journal.pone.0001504)
Supplement: Table S6 — Direction of expression differences among the 117 diet-related genes. (0.03 MB DOC) [file pone.0001504.s006.doc]

|  | | Human diet *versus* chimpanzee diet in mouse ^b^ | |
| --- | --- | --- | --- |
|  |  | **+** | **-** |
| Human  *versus*  chimpanzee ^a^ | **+** | 51 | 14 |
|  | - | 41 | 11 |

**^a^** Number of genes with positive/negative effect sizes in the comparison between human and chimpanzee livers (a positive effect size indicates higher expression in humans).

**^b^** Number of genes with positive/negative effect sizes in the comparison between mice fed human and chimpanzee diets in liver (a positive effect size indicates higher expression in mice fed the human diets).
