# Supplementary material for: Human and Chimpanzee Gene Expression Differences Replicated in Mice Fed Different Diets
Source: PLoS One. 2008 Jan 30;3(1):e1504. doi: 10.1371/journal.pone.0001504 (PMC2200793; doi:10.1371/journal.pone.0001504)
Supplement: Table S7 — Expression divergence on the human versus the chimpanzee lineage among the 117 diet-related genes. (0.03 MB DOC) [file pone.0001504.s007.doc]

| **Control set** | **# Diet-**  **related**  **genes ^a^** | **# Control genes ^b^** | **Mann Whitney U test *p*-value^c^** | **Permutation test *p*-value^c^** | **Median of diet-related genes^d^** | **Median of control genes^d^** |
| --- | --- | --- | --- | --- | --- | --- |
| All diff. | 117 | 1035 | 0.059 | 0.047 | 2.20 | 1.76 |
| All det. | 117 | 6900 | 4E-6 | 0.001 | 2.20 | 0.80 |
| All genes | 117 | 10449 | 4E-7 | 0.001> | 2.20 | 0.65 |

**^a^** Human genes with mouse orthologs showing diet-related human-chimpanzee expression differences in liver.

**^b^** The three sets of non-diet-related genes that are compared with diet-related genes. These are: **All diff. -** Human genes with mouse orthologs showing human-chimpanzee expression differences in liver. **All det. -** Human genes with mouse orthologs expressed in liver. **All genes -** All available human genes with mouse orthologs.

**^c^** See Materials and Methods for a description of the applied test.

**^d^** The median value of expression divergence on the human versus chimpanzee lineages for the relevant gene set. Values larger than 1 indicate higher divergence on the human lineage. See Materials and Methods for details.
