# Supplementary material for: Human and Chimpanzee Gene Expression Differences Replicated in Mice Fed Different Diets
Source: PLoS One. 2008 Jan 30;3(1):e1504. doi: 10.1371/journal.pone.0001504 (PMC2200793; doi:10.1371/journal.pone.0001504)
Supplement: Table S8 — Sequence divergence patterns among the 117 diet-related genes. (0.04 MB DOC) [file pone.0001504.s008.doc]

|  | **Control set** | **# Diet-**  **related**  **genes ^a^** | **# Control genes ^b^** | **Mann Whitney U test *p*-value^c^** | **Permutation test *p*-value^c^** | **Median of diet-related genes^d^** | **Median of control genes^d^** |
| --- | --- | --- | --- | --- | --- | --- | --- |
| **Human-Chimpanzee Promoter Divergence** | All diff. | 70 | 566 | 0.013 | 0.010 | 0.011 | 0.010 |
|  | All det. | 70 | 3976 | 0.006 | 0.006 | 0.011 | 0.010 |
|  | All genes | 70 | 7995 | 0.009 | 0.004 | 0.011 | 0.010 |
|  | | | | | | | |
| **Human-Chimpanzee Amino Acid Divergence (Ka/Ki)** | All diff. | 72 | 634 | 0.002 | 0.002 | 0.183 | 0.110 |
|  | All det. | 72 | 4380 | 3E-4 | 0.001> | 0.183 | 0.098 |
|  | All genes | 72 | 9321 | 0.067 | 0.043 | 0.183 | 0.143 |

**^a^** Human genes with mouse orthologs showing diet-related human-chimpanzee expression differences in liver, for which a divergence estimate exists.

**^b^** The three sets of non-diet-related genes that are compared with diet-related genes in terms of rates of evolution. These are: **All diff. -** Human genes with mouse orthologs showing human-chimpanzee expression differences in liver. **All det. -** Human genes with mouse orthologs expressed in liver. **All genes -** All available human genes with mouse orthologs.

**^c^** See Materials and Methods for a description of the applied tests.

**^d^** The median promoter divergence or amino acid divergence values for the relevant gene set.
