# Supplementary figures and images for: Human and Chimpanzee Gene Expression Differences Replicated in Mice Fed Different Diets
Source: PLoS One. 2008 Jan 30;3(1):e1504. doi: 10.1371/journal.pone.0001504 (PMC2200793; doi:10.1371/journal.pone.0001504)

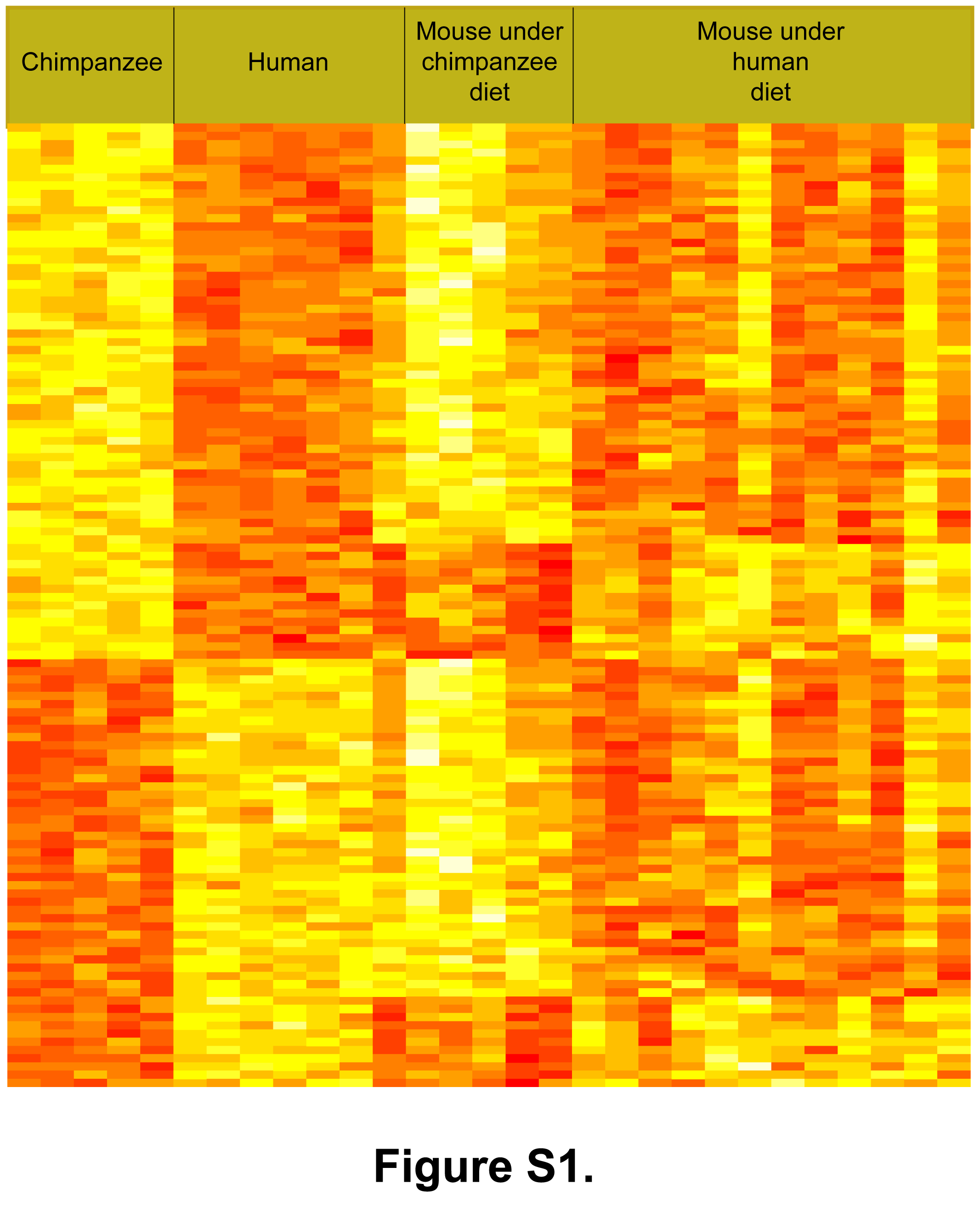

Supplement: Figure S1 — Heatmap of expression patterns of the 117 diet-related genes. Each row represents a gene, each column a sample: Either gene expression levels in the livers of mice fed human or chimpanzee diets, or in the livers of humans or chimpanzees (from left to right). Expression values higher than the average for each gene are represented in red, values lower than the average in yellow and white. (0.26 MB TIF) [file pone.0001504.s010.tif]

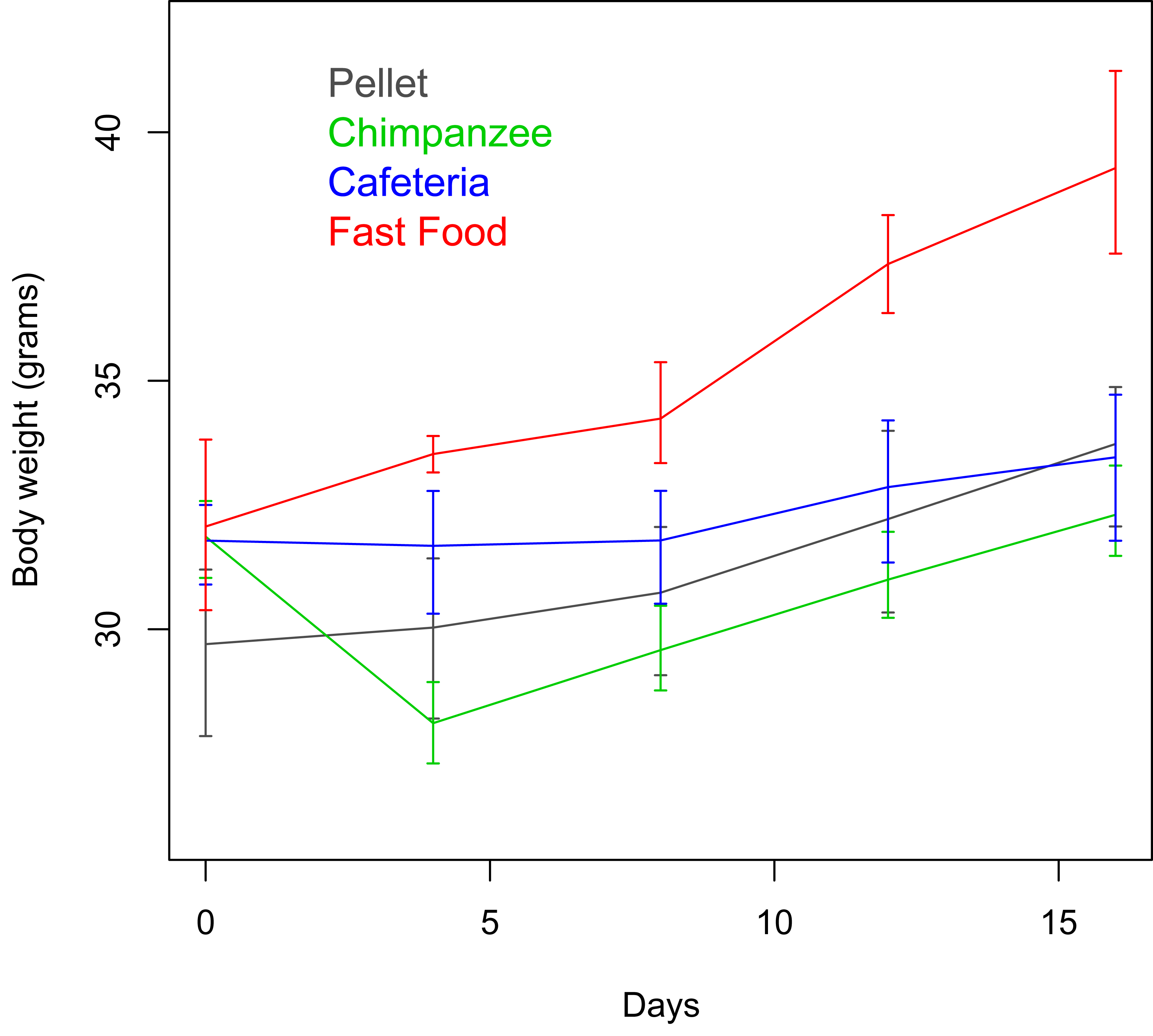

Supplement: Figure S2 — Weight changes in mice fed different diets. The y-axis indicates the mean body weight among mice fed one of four different diets, measured at four days intervals during the experiment. Error bars show 95% confidence intervals of the mean based on 1,000 bootstraps. Pellet: The mouse pellet diet. Chimpanzee: The diet fed to chimpanzees in the Leipzig zoo. Cafeteria: The MPI-EVA Cafeteria diet. Fast Food: A pure McDonald's diet. (0.27 MB TIF) [file pone.0001504.s011.tif]
